# Are neuropsychiatric symptoms a marker of small vessel disease progression in older adults? Evidence from the Lothian Birth Cohort 1936

**DOI:** 10.1002/gps.5855

**Published:** 2022-12-09

**Authors:** Una Clancy, Ratko Radakovic, Fergus Doubal, Maria del C. Valdés Hernández, Susana Muñoz Maniega, Adele M. Taylor, Janie Corley, Francesca M. Chappell, Tom C. Russ, Simon R. Cox, Mark E. Bastin, Ian J. Deary, Joanna M. Wardlaw

**Affiliations:** ^1^ Centre for Clinical Brain Sciences University of Edinburgh Edinburgh UK; ^2^ UK Dementia Research Institute at The University of Edinburgh Edinburgh UK; ^3^ Department of Clinical Psychology and Psychological Therapies University of East Anglia Norwich UK; ^4^ Lothian Birth Cohorts Department of Psychology University of Edinburgh Edinburgh UK; ^5^ Alzheimer Scotland Dementia Research Centre University of Edinburgh Edinburgh UK; ^6^ Euan MacDonald Centre for MND Research University of Edinburgh Edinburgh UK; ^7^ Scottish Imaging Network, a Platform for Scientific Excellence (SINAPSE) Collaboration Edinburgh UK; ^8^ Division of Psychiatry Centre for Clinical Brain Sciences University of Edinburgh Edinburgh UK

**Keywords:** ageing, apathy, cerebral small vessel disease, cognition, longitudinal studies, white matter hyperintensities

## Abstract

**Background:**

Neuropsychiatric symptoms could form part of an early cerebral small vessel disease prodrome that is detectable before stroke or dementia onset. We aimed to identify whether apathy, depression, anxiety, and subjective memory complaints associate with longitudinal white matter hyperintensity (WMH) progression.

**Methods:**

Community‐dwelling older adults from the observational Lothian Birth Cohort 1936 attended three visits at mean ages 73, 76, and 79 years, repeating MRI, Mini‐Mental State Examination, neuropsychiatric (Dimensional Apathy Scale, Hospital Anxiety and Depression Scale), and subjective memory symptoms. We ran regression and mixed‐effects models for symptoms and normalised WMH volumes (cube root of WMH:ICV × 10).

**Results:**

At age 73, 76, and 79, *m* = 672, *n* = 476, and *n* = 382 participants attended MRI respectively. Worse apathy at age 79 was associated with WMH volume increase (*β* = 0.27, *p* = 0.04) in the preceding 6 years. A 1SD increase in apathy score at age 79 associated with a 0.17 increase in WMH (*β* = 0.17 normalised WMH percent ICV, *p* = 0.009). In apathy subscales, executive (*β* = 0.13, *p* = 0.05) and emotional (*β* = 0.13, *p* = 0.04) scores associated with increasing WMH more than initiation scores (*β* = 0.11, *p* = 0.08). Increasing WMH also associated with age (*β* = 0.40, *p* = 0.002) but not higher depression (*β* = ‐0.01, *p* = 0.78), anxiety (*β* = 0.05, *p* = 0.13) scores, or subjective memory complaints (*β* = 1.12, *p* = 0.75).

**Conclusions:**

Apathy independently associates with preceding longitudinal WMH progression, while depression, anxiety, and subjective memory complaints do not. Patients with apathy should be considered for enrolment to small vessel disease trials.

## Key points

- Worse apathy at age 79 associates with greater progression of white matter hyperintensities (WMH) in the preceding 6 years.
- In this population of community‐dwelling older adults, WMH progression is not associated with anxiety, depression, or subjective memory complaints.
- Apathy has potential as a key clinical marker of SVD progression.

## 1 INTRODUCTION

Cerebral small vessel disease (SVD) is highly prevalent in older people and is a common cause of vascular dementia and stroke.^1^ It is not clear whether early symptoms of SVD could predict the development of these conditions. Identifying an ‘SVD syndrome’ would allow clinicians and researchers to identify individuals who might benefit from future clinical trials or earlier SVD preventative interventions.^2^

Neuropsychiatric and cognitive symptoms have been proposed as potential candidates for early clinical detection of SVD. Recent work has established that cross‐sectional relationships exist between these symptoms and worse white matter hyperintensity (WMH) burden,^3^, ^4^ a key radiological feature of SVD.^5^ Specifically, more severe WMH burden associates with apathy and depression. In contrast, more severe WMH burden does not associate cross‐sectionally with subjective memory complaints (SMCs), while associations with anxiety and other neuropsychiatric symptoms are indeterminate.^3^, ^4^ Longitudinal imaging‐symptoms studies are required to confirm these associations. However, such studies are sparse (online supplemental Table 1).^6^, ^7^, ^8^, ^9^, ^10^, ^11^

We aimed to determine whether anxiety, depression, apathy, or SMCs are each associated with longitudinal WMH volume change in an older community‐dwelling cohort over 6 years.

## 2 MATERIALS AND METHODS

### 2.1 Participants

**FIGURE 1:**
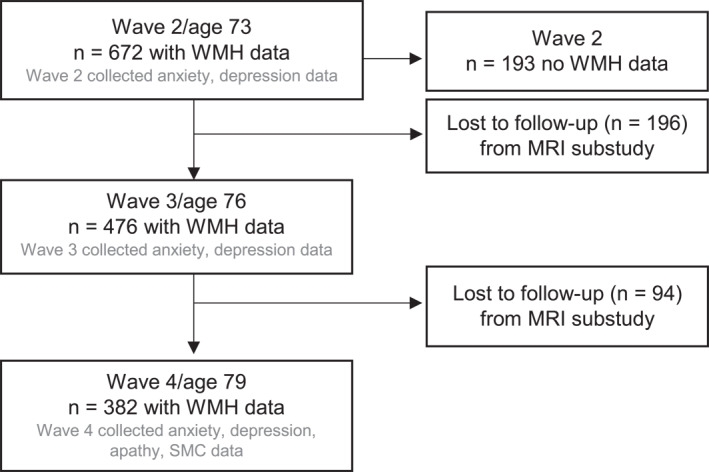
Flow chart of study participants.

This analysis consists of individuals in the Lothian Birth Cohort 1936 (LBC1936), an ongoing longitudinal ageing study. The LBC1936 comprises older adults born in 1936, most of whom took the Scottish Mental Survey of 1947 aged 11 years. Almost 60 years after first taking the Scottish Mental Survey of 1947, surviving participants of the survey were identified via local health board records and invited to take part in LBC1936 by post. Eligible participants who responded and consented were recruited.^12^ The only MRI substudy exclusion criterion was contraindication to MRI, for example, metal implant, claustrophobia. Community‐dwelling individuals living in Edinburgh and the surrounding areas attended the first LBC1936 study wave (total *n* = 1091) from 2004 to 2007 at mean 70 years old. Participants then attended the second (total *n* = 866), third (total *n* = 697), and fourth waves (total *n* = 550) at mean ages of 73, 76, and 79, respectively. Participants were invited to MRI scans, which were first conducted at Wave two. The present analysis focuses on Waves two to four, that is, individuals at mean ages of 73, 76, and 79 years at study visits. The protocol^12^ and population profiles are published in detail.^13^, ^14^ Figure 1 shows the study flow chart.

The cognitive status of the LBC1936 has been described previously and applies to 397/476 (83%) of Wave three and 319/382 (84%) of Wave four participants included in the present analysis.^15^, ^16^ The majority did not have cognitive impairment and none of the participants reported a dementia diagnosis at recruitment. Three participants had had a dementia diagnosis by Wave three. In participants with relevant data available to make a mild cognitive impairment (MCI) diagnosis,^17^ MCI was present in 15% at Wave three and 17% at Wave four.^15^

### 2.2 Neuropsychiatric and cognitive symptom assessments

All symptoms were assessed using self‐completed questionnaires. Depression and anxiety were assessed using the Hospital Anxiety and Depression Scale^18^ at each wave (score ranges 0–21).

Apathy was assessed using the Dimensional Apathy Scale (DAS)^19^ at age 79 (Wave four) only. The DAS is a multidimensional scale which assesses different apathy subtypes^19^ based on subtype relationships to damage and impaired connectivity in the pre‐frontal cortico‐subcortical and basal ganglia regions.^20^ Subscale analysis of the DAS provides further insight into global apathy by looking at specific characteristics of demotivation as follows. Executive apathy is a lack of motivation for planning, organisation and attention. Emotional apathy is a lack of emotional motivation, indifference, emotional neutrality, blunting or flatness. Initiation apathy is a lack of motivation for self‐generated thoughts and/or behaviours. The total score ranges from 0 to 72 and the subscales from 0 to 24. Although there is no predefined cut‐off for the DAS, normative data have previously been used to suggest abnormality level cut‐offs for each subscale based on ≥2 SD above the mean.^21^ We did not use a cut‐off for the present analysis. Higher scores indicate worse apathy.

The present analysis includes three questions about SMCs which were asked at age 79 (Wave four) only: Q1 “Do you currently have any problems with your memory?”, Q2 “If yes, are these problems interfering with your normal life?”, and Q3 “Do you forget where you have left things more often than you used to?”. We describe each symptom individually but use Question 1 in multivariable analyses, to maintain consistency with the existing SMC literature.

### 2.3 Clinical assessments

Self‐reported years of education, that is, full‐time schooling, were collected at Wave one. The following measures were collected at all waves. Self‐reported vascular risk factor diagnoses were recorded, that is, binary history (yes/no) of cardiovascular disease, diabetes, hypercholesterolaemia, hypertension, stroke history, and smoking status (current/ex/never). We summed a composite vascular risk factor score containing these variables, described previously,^22^ to avoid model overfitting. Research nurses completed the Townsend disability scale, a measure of activities of daily living.^23^ This cohort had comprehensive multidomain cognitive assessments,^24^, ^25^ but since cognition was not a main outcome of interest in the present analysis, we used the Mini‐Mental State Examination (MMSE),^26^ a test of global cognitive function commonly used as a screening test for cognitive impairment. This was interviewer‐administered at each wave. We included cognition to adjust for neuropsychiatric symptom associations.^27^

### 2.4 MRI acquisition

Participants were invited to attend MRI at mean ages73, 76, and 79 (Waves two, three, and four), performed on the same 1.5 T scanner (Signa Horizon HDx; General Electric, Milwaukee, WI). Acquisition parameters are detailed in full elsewhere^28^ but included T1‐ (slice thickness 1.3 mm; voxel resolution 1 × 1 × 1.3 mm),T2‐ (thickness 2 mm; resolution 1 × 1 × 2 mm) and T2*‐weighted (thickness 2 mm; resolution 1 × 1 × 2 mm), FLAIR (thickness 4mm; resolution 1 × 1 × 4 mm), diffusion tensor (thickness 2mm; resolution 2 × 2 × 2 mm), T1 mapping (thickness 2 mm; resolution 1 × 1 × 2 mm), and magnetization transfer (thickness 2 mm; resolution 1 × 1 × 2 mm) sequences using a self‐shielding gradient with maximum strength 33 mT/m.^28^

### 2.5 MRI analysis

We combined well‐validated computational methods with manual checking to quantify WMH volumes, per the Standards for ReportIng Vascular Changes on Neuroimaging (STRIVE) guidelines.^5^ Quantitative analysis methods are detailed elsewhere.^28^, ^29^ In brief, we used a semi‐automated multispectral segmentation tool to extract volumes including WMH and intracranial volume (ICV), co‐registering T1‐, T2‐, T2*‐weighted and FLAIR images to the T2‐weighted images using FSL‐FLIRT. This highly reproducible method, complemented by manually checking all images, includes distinction between WMH and chronic infarcts and is overseen by an experienced neuroradiologist (JMW). Image analysts and neuroradiologists were blinded to all non‐imaging data. Full structural brain characteristics for the cohort are described elsewhere.^30^

Ethical approval was granted by the Multi‐Centre Research Ethics Committee for Scotland (Wave one: MREC/01/0/56), the Lothian Research Ethics Committee (Wave one: LREC/2003/2/29), and the Scotland A Research Ethics Committee (Waves two/three/four: 07/MRE00/58). Participants gave written informed consent.

### 2.6 Statistical analysis

We compared participants who did or did not attend (a) the first MRI visit and (b) follow‐up MRI visits. We expressed WMH volume as a ratio of ICV and calculated the cube root to optimise model fit and minimise model complexity, as described previously,^31^ multiplying all values by 10 to scale with other variables. We created a sum vascular risk factor score comprising hypertension, hyperlipidaemia, smoking status, and diabetes, assigning equal weight to the presence of each factor, as described previously.^32^ The rationale for this was to include relevant variables while avoiding model overfitting. For descriptive symptom analyses, we calculated Pearson (*r*) and Spearman (rs) correlation coefficients and Wilcoxon rank‐sum tests (*W*).

We assessed associations between WMH and each neuropsychiatric or cognitive symptom as a dependent variable to explore whether SVD lesions associate with neuropsychiatric and cognitive symptoms. We describe data collection timelines in Figure 1.

First, to assess cross‐sectional symptom‐WMH associations at each wave, we ran separate linear regression models using Wave two (anxiety, depression), three (anxiety, depression), and four (anxiety, depression, apathy, SMCs) data. Then, to assess longitudinal anxiety and depression associations with WMH, we ran separate linear mixed‐effects models with each symptom as the outcome and normalised WMH volume (expressed as cube root of WMH:ICV [×10]) as the exposure, using data from all three time‐points. We adjusted for time (i.e. wave number), age at each scan, MMSE, sex, anxiety/depression, vascular risk factors, and years of education. We additionally adjusted for disability in depression models to account for depression‐disability associations.^33^

Since apathy and SMCs were collected at age 79 (Wave four) only, the longitudinal assessment analysis of these symptoms required a different statistical approach which assessed preceding WMH volume change. For each model, we defined WMH volume change as the difference between age 73 (Wave two) and age 79 (Wave four) WMH volumes. We ran linear regression (apathy) and generalised linear models (SMCs) with each symptom as the outcome and assessed associations with WMH volume change. We corrected for baseline WMH volumes, MMSE, depression scores, activities of daily living, vascular risk factor scores, age, sex, and education.

In our final set of analyses we reversed the research question, using WMH as the outcome and neuropsychiatric symptoms as the predictors. All neuropsychiatric symptoms (apathy, subjective memory complaints, anxiety, depression) were included as predictors in this final model (online supplemental Figure 8).

Since longitudinal research assessing neuropsychiatric symptoms and SVD progression is a relatively new area of exploration in the SVD‐neuropsychiatric symptom literature,^3^ we regard this as an exploratory analysis. Therefore, to avoid the probability of a Type II error,^34^ we planned all comparisons prior to data analysis, set alpha level to 0.05, reported all analyses performed, and did not correct for multiple comparisons so that any given *p* value can be interpreted based on the analysis in question.^35^ We present fully adjusted models defined according to our clinical hypothesis, adjusted for all clinically relevant covariates supported by previous literature.

We report standardised beta (*β*) coefficients. For all mixed‐effects models, we fitted individual participants as the random effect to assess variation within individuals, using R package lme4.^36^

## 3 RESULTS

### 3.1 Baseline population characteristics

We include *n* = 672 participants who attended MRI at mean age 73 (Wave two), *n* = 476 at mean age 76 (Wave three), and *n* = 382 at mean age 79 (Wave four) (Figure 1). At Wave two, there were no differences in age, sex, vascular risk factor scores, disability, MMSE, anxiety, or depression among participants who did (*n* = 672) versus did not (*n* = 193) take part in the optional MRI sub‐study (online supplemental Table 2). Similarly, participants lost to MRI follow‐up were no different to participants who attended all three MRI visits (online supplemental Table 2). Reasons for attrition are published elsewhere.^14^

At baseline, included participants were mean 72.7 (SD 0.72) years old; 47% female; with median MMSE score of 29 (IQR 28–30), and disability scores of 0 (IQR 0–1). WMH volumes increased by mean 8.43ml (SD 8.74; range −10.4 to 51.7) between ages 73 and 79 (Waves two and four; online supplemental Figure 1; online supplemental Figure 2). Table 1 shows population characteristics at each wave.

**TABLE 1:** Population characteristics of participants who attended MRI at waves two, three, or four

	Wave 2 (*N* = 672)	Wave 3 (*N* = 476)	Wave 4 (*N* = 382)
Age (years); mean (SD)	72.7 (0.7)	76.4 (0.6)	79.5 (0.6)
Female	316 (47.0%)	222 (46.6%)	179 (46.9%)
Full‐time education (years); mean (SD)	10.8 (1.13)	10.8 (1.15)	10.9 (1.19)
Cardiovascular disease	182 (27.1%)	160 (33.6%)	139 (36.4%)
Diabetes	69 (10.3%)	59 (12.4%)	50 (13.1%)
Hypercholesterolaemia	283 (42.1%)	228 (47.9%)	184 (48.2%)
Hypertension	331 (49.3%)	261 (54.8%)	220 (57.6%)
History of stroke	46 (6.8%)	54 (11.3%)	53 (13.9%)
Smoking status: Never	318 (47.3%)	243 (51.1%)	205 (53.7%)
Smoking status: Ex‐smoker	300 (44.6%)	203 (42.6%)	164 (42.9%)
Smoking status: Current	54 (8.0%)	29 (6.1%)	13 (3.4%)
Townsend's disability scale score, median [Q1, Q3]	0 [0, 1]	0 [0, 2]	0 [0, 2]
MMSE score, Median [Q1, Q3]	29 [28, 30]	29 [28, 30]	2 [28, 30]
HADS Anxiety score median [Q1, Q3]	4 [2, 6]	4 [2, 6]	3 [2, 6]
HADS Depression score, median [Q1, Q3]	2 [1, 4]	2 [1, 4]	2 [1, 4]
Total Dimensional Apathy Scale, mean (SD)	‐	‐	25 (7.59)
Executive apathy Mean (SD)			5.9 (3.6)
Emotional apathy, mean (SD)			8.9 (2.9)
Initiation apathy, Mean (SD)			10.3 (4.2)
Do you currently have any problems with your memory: Yes	‐	‐	214 (62.2%)
If yes, are these problems interfering with your normal life: Yes	‐	‐	16 (4.7%)
Do you forget where you left things more than you used to: Yes	‐	‐	170 (49.4%)
WMH volume (ml), mean (SD)	12.1 (12.8)	16.3 (15.6)	19.8 (17.3)
WMH volume (ml) Median [Q1, Q3]	7.7 [3.6, 17.0]	11.3 [5.4, 22.9]	14.6 [7.3, 26.6]
Deep atrophy rating mean (SD)	3.55 (1.28)	3.27 (1.39)	‐
Superficial atrophy rating, Mean (SD)	3.54 (1.23)	3.26 (1.29)	‐
Periventricular Fazekas WMH rating, Mean (SD)	1.35 (0.64)	1.47 (0.71)	‐
Deep Fazekas WMH rating, mean (SD)	1.09 (0.66)	1.16 (0.67)	‐

Abbreviations: HADS, Hospital Anxiety and Depression Scale; MMSE, Mini‐mental state examination; Q1, quartile one; Q3, quartile three; SD, standard deviation; WMH, white matter hyperintensities.

### 3.2 Neuropsychiatric and cognitive symptom characteristics

At baseline (age 73), the median depression score was 2 (1–4; range 0–15) and the median anxiety score was 4 (IQR 2–6; range 0–19). At age 79 (Wave four), the mean total apathy score was 25 (SD 7.59; range 5–48). DAS subtype scores are shown in Table 1. SMCs were reported in 62.2% but rarely interfered with normal life (4.7%).

At age 79, depression scores were positively correlated with total apathy (*r* = 0.47, *p* < 0.001) and with each of the apathy subtypes: executive (*r* = 0.56, *p* < 0.001); emotional (*r* = 0.17, *p* = 0.003); and initiation apathy (*r* = 0.37, *p* < 0.001). Depression scores were also positively correlated with anxiety scores (rs = 0.33, *p* < 0.001). Anxiety positively correlated with total apathy (rs = 0.23 *p* < 0.001) and with the executive apathy subtype (rs = 0.41, *p* < 0.001), but not with emotional (rs = −0.03, *p* = 0.62) or initiation apathy subtypes (rs = 0.02, *p* = 0.70). SMCs were associated with higher depression (*W* = 26,619, *p* < 0.001) and apathy (*W* = 6955, *p* = 0.02), but not anxiety scores (*W* = 31,561, *p* = 0.17). Between ages 73 to 79, depression scores increased by a mean of 0.48 (SD 1.97) points and anxiety scores decreased by a mean of 0.24 (SD 2.54) points (online supplemental Figure 1).

### 3.3 WMH associations with neuropsychiatric and cognitive symptoms

#### 3.3.1 WMH change as predictor and wave 2/3/4 depression as outcome

There were no cross‐sectional associations between depression scores and WMH volumes (cube root of WMH:ICV [×10]) at any visit (at Wave four, *β* = −0.02, *p* = 0.67; online supplemental Table 3/online supplemental Figure 3A). In longitudinal mixed‐effects analysis (*n* = 688), depression scores were not associated with WMH volume changes (*β* = −0.01, *p* = 0.78; online supplemental Table 4‐5/online supplemental Figure 3B).

#### 3.3.2 WMH change as predictor and wave 2/3/4 anxiety as outcome

In cross‐sectional analyses, anxiety scores were not associated with WMH volumes at any wave (at Wave four, *β* = −0.01, *p* = 0.82; online supplemental Table 6/online supplemental Figure 4A). Longitudinal mixed‐effects analysis (*n* = 689) produced similar results, that is, anxiety scores were not associated with changing WMH volumes (*β* = 0.05, *p* = 0.13; online supplemental Figure 4B/online supplemental Table 7).

#### 3.3.3 WMH change as predictor and wave 4 apathy as outcome

**FIGURE 2:**
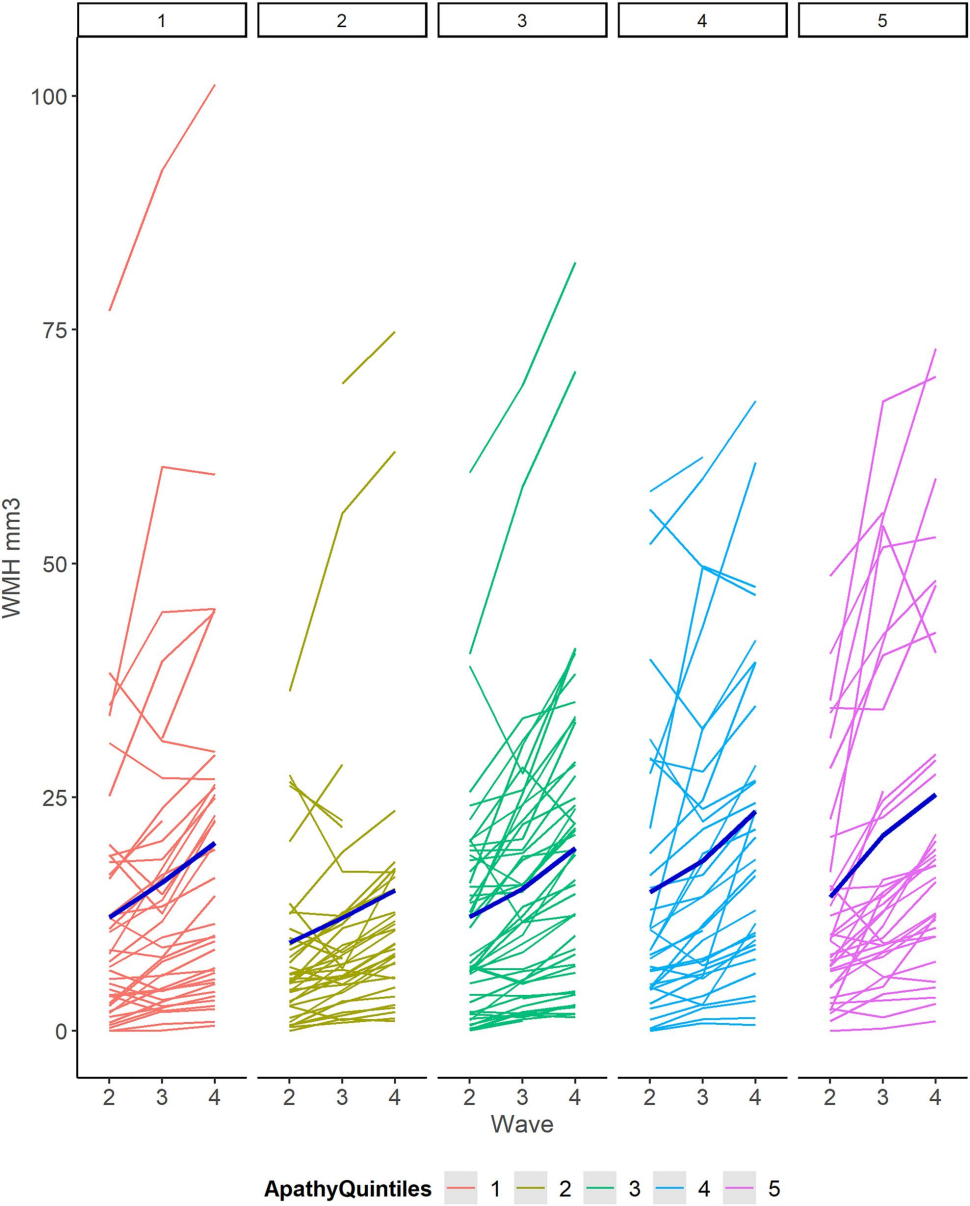
Individual white matter hyperintensities (WMH) volume change at Waves 2/3/4 according to quintiles of apathy severity (Wave 4). Quintile 1 = lowest apathy scores; quintile 5 = highest apathy scores.

In cross‐sectional analysis (*n* = 197), there was a small trend towards an association between higher apathy scores and WMH volumes at the age of 79 (Wave four; online supplemental Figure 6). In linear regression analysis (*n* = 191), higher age 79 (Wave four) total apathy scores were associated with greater WMH volume increases between ages 73 to 79 (over the preceding six years; *β* = 0.27, *p* = 0.04), adjusting for baseline WMH volume, male sex, baseline depression, and depression score change (Table 2 and Figure 2).

**TABLE 2:** Linear regression: Associations between apathy and change in WMH volume, depression, MMSE and disability scores between waves two and four

Predictors	Apathy score wave four		
	std. Beta	Standardized CI	*p*
(Intercept)	0.24	0.09 to 0.40	0.986
Age (years)	0.01	−0.11 to 0.13	0.887
Sex: Female	−0.52	−0.75 to −0.29	<0.001
MMSE score change	−0.08	−0.20 to 0.03	0.163
Depression score change	0.46	0.33 to 0.60	<0.001
Baseline Depression score	0.53	0.39 to 0.66	<0.001
Normalised WMH volume: ICV change	0.27	0.01 to 0.54	0.043
Baseline Normalised WMH volume: ICV	−0.11	−0.37 to 0.16	0.426
Vascular risk factors	0.01	−0.10 to 0.13	0.829
Townsend Disability score change	0.05	−0.07 to 0.17	0.419
Years of education	0.06	−0.05 to 0.18	0.275

*Note*: Observations = 191; *R* ^2^/*R* ^2^ adjusted = 0.410/0.378.

#### 3.3.4 WMH change as predictor and wave 4 SMCs as outcome

Self‐reported memory problems (SMC Question 1) were not associated with preceding WMH volume changes (*β* = 1.12, *p* = 0.75) (online supplemental Table 8/online supplemental Figure 7).

#### 3.3.5 Alternative hypothesis: Symptoms as predictors and WMH progression as outcome

Individuals in the quintile of greatest WMH increase between the ages of 73 and 79 had higher apathy scores at age 79 (mean 27.64 [SD 7.35]) than individuals in the lowest quintile (mean 24.78 [SD 7.43]). In mixed‐effects analysis of all three waves, a one standard deviation higher increment in apathy score at age 79 (Wave four) was associated with a preceding 0.17 increase in normalised WMH volumes between Waves two and four (*β* = 0.17, *p* = 0.009; Table 3/online supplemental Figure 8). Higher scores on the emotional (*β* = 0.13, *p* = 0.04) and executive DAS subscales (*β* = 0.13, *p* = 0.05) had slightly stronger associations with WMH volume change than the initiation subscale (*β* = 0.11, *p* = 0.08; online supplemental Table 9/online supplemental Figure 9). Higher scores on the combined emotional plus executive DAS subscales (*β* = 0.18, *p* = 0.006) had a stronger association with WMH volume change than initiation plus emotional (*β* = 0.14, *p* = 0.02) or initiation plus executive (*β* = 0.14, *p* = 0.02) combined subscales. There was no association between vascular risk factor scores and WMH, adjusted for age (Table 3, online supplemental Figure 8). Associations between apathy and WMH increase remained after adjusting for stroke history (online supplemental Figure 8).

**TABLE 3:** Linear mixed‐effects model: Factors associated with WMH volume changes across three waves

Predictors	Normalised WMH volume: ICV		
	std. Beta	Standardized CI	*p*
(Intercept)	−0.03	−0.27 to 0.20	0.019
Wave	−0.17	−0.43 to 0.08	0.181
Age (years)	0.40	0.15 to 0.66	0.002
Sex: Female	0.09	−0.16 to 0.35	0.479
MMSE	−0.03	−0.06 to 0.00	0.091
Anxiety	0.03	−0.02 to 0.07	0.278
Depression score	−0.03	−0.07 to 0.02	0.214
Apathy score total	0.17	0.04 to 0.30	0.009
Subjective memory complaints (Q1): Yes	0.00	−0.26 to 0.26	0.979
Vascular risk factors	−0.01	−0.06 to 0.05	0.781
Townsend's disability scale score	−0.01	−0.05 to 0.03	0.714
Years of education	−0.01	−0.13 to 0.11	0.878

*Note*: Random effects *σ* ^2^ = 0.03, *τ*_00_ _lbc36no_ = 0.44, ICC = 0.93. N _lbc36no_ = 226; Observations = 596; Marginal *R* ^2^/Conditional *R* ^2^ = 0.082/0.934.

## 4 DISCUSSION

The present study indicates that higher apathy scores at age 79 are associated with greater WMH progression over time in adults aged 73–79, independent of age, sex, vascular risk factors, disability, and education. WMH volumes increased by a mean of 8.43ml (i.e. 1 and 1/3 of a teaspoon), with a range of −10.4 to 51.7ml between the ages of 73 and 79 years in this community‐dwelling, relatively healthy cohort. Depression, anxiety, and SMCs are not associated with WMH presence or progression. It is biologically plausible that apathy could arise from direct structural damage to white matter mediated connections. For example, post‐stroke apathy is a known complication of infarcts affecting the basal ganglia. We highlight apathy as a potentially informative marker of SVD progression, beyond established markers for which we adjusted. Our deeper analysis of apathy subtypes suggests that different subtypes have different degrees of association related to WMH outcome, that is, executive and emotional apathy play stronger roles than initiation apathy.

The LBC1936 is a well‐characterised cohort with rich longitudinal data, allowing adjustment for key confounders. The analysis had limitations.We did not assess the full spectrum of SVD‐related pathology. Further work is needed to establish how other structural features (e.g. perivascular spaces, lacunes) and subvisible abnormalities (e.g. on diffusion imaging) manifest clinically across different locations, stages of development, and severity.^37^, ^38^ We focused on total WMH volumes rather than specific regional networks,^39^ which may have limited the sensitivity for detecting imaging associations of symptoms. However, since SVD is a diffuse disease, we suggest that total WMH volume is still a relevant global measure of overall SVD burden. Our use of a summary vascular risk factor score, which has been used in other studies to avoid model overfitting, may have underestimated relationships between vascular risk and WMH. Unlike anxiety and depression, apathy and SMCs were only collected at Wave four: data for these symptoms from earlier waves would have allowed us to conduct lead‐lag analyses to understand the temporal order of these symptoms and WMH. Therefore, the study design did not allow us to investigate whether brain changes lead to greater apathy or the vice versa. Although we transformed WMH volumes as the cube root of WMH:ICV [×10] for optimal statistical analysis, this makes the model beta coefficients more difficult to interpret clinically, but our results do show the direction of association and significance. We did not correct for multiple comparisons and our results should be interpreted in the context of the large number of comparisons carried out and the lack of statistical independence among variables, but we openly report results of all analyses performed and any given *p* value can be interpreted based on the analysis in question.^35^ This is an exploratory analysis and the findings should be cautiously interpreted and replicated in larger samples.

Our results extend evidence beyond cross‐sectional apathy associations with WMH,^3^ while adjusting for depression. We confirm the absence of a WMH‐anxiety association suggested in previous cross‐sectional work,^3^ by using continuous WMH volumes in a large longitudinal sample. Our findings agree with and build on a previous study of SMCs,^10^ enhanced by our analysis of WMH volumes as a continuous variable. Four previous studies have assessed WMH volumes longitudinally with depression.^6^, ^7^, ^8^, ^9^ Our finding of no longitudinal WMH‐depression association agrees with findings from one study of adults >70 years^8^ but differs from three others (online supplemental Table 1).^6^, ^7^, ^9^ This may be due to differences in adjustment for confounders (especially apathy), duration of follow‐up, number of imaging time‐points, methods of analysing intraindividual differences, measurement scales, or attrition of depressed patients from previous studies. Our findings are consistent with the high prevalence of apathy in genetic forms of SVD, for example, CADASIL.^40^ The broader range of mild behavioural and psychological impairments that frequently herald MCI and dementia require further study in relation to SVD progression.^3^

A one standard deviation increment in apathy score at age 79 was associated with a 0.17 increase in normalised WMH volumes in the preceding 6 years, and at age 79, individuals with the greatest preceding WMH volume increase scored three apathy points higher than individuals who had had the smallest increase in WMH volumes. This represents a small but potentially important and recognisable association, motivating further work into the role of apathy as a clinical marker of SVD. Depression per se, anxiety, and SMCs require further assessment in a range of populations.

### 4.1 Future directions for SVD symptom‐lesion associations

We have highlighted a key timeframe in older age during which SVD progresses rapidly, with WMH almost doubling over 6 years, representing a vital window of opportunity to target potential treatments. It is too early to say whether apathy scores would be useful on an individual basis, but further research into clinical prediction models is needed to guide whether treatments could be trialled in individuals who are identified as being at the highest risk for WMH progression, that is, older adults with apathy. Longitudinal apathy analyses would be useful to see whether apathy change, versus apathy at one time‐point, could be more useful to track SVD progression. It remains to be seen whether symptoms precede the detectable appearance of SVD lesions, clinically reflecting the early stages of pre‐lesion pathology.

Symptom‐lesion associations need to be explored at varying time intervals, assessing acute, subacute, and chronic brain changes. Our research question should also be extended to other non‐focal symptoms that are not currently sufficient to contribute to a stroke or dementia diagnosis.

The findings from this study will assist in identifying an early SVD syndrome and encourage us to broaden our focus beyond stroke and dementia. Apathy may help to distinguish SVD ‘progressors’ from ‘non‐progressors’ in the general population. We need to accelerate efforts to uncover other subtle symptoms of SVD progression. This will identify an as‐yet underrepresented population who could benefit from early targeted potential SVD treatments.

## AUTHOR CONTRIBUTIONS

Drafting/revision of the manuscript; (all authors); Analysis or interpretation of data (Una Clancy, Ratko Radakovic, Francesca M. Chappell, Simon R. Cox, Ian J. Deary, Joanna M. Wardlaw); Major role in acquisition of data (Maria del C. Valdés Hernández, Susana Muñoz Maniega, Adele M. Taylor, Janie Corley, Ian J. Deary, Joanna M. Wardlaw); Study concept or design (Ian J. Deary, Joanna M. Wardlaw).

## CONFLICT OF INTEREST

None declared.

## ETHICS STATEMENT

This study received ethical approval from the Multi‐Centre Research Ethics Committee for Scotland (Wave one: MREC/01/0/56), the Lothian Research Ethics Committee (Wave one: LREC/2003/2/29), and the Scotland A Research Ethics Committee (Waves two/three/four: 07/MRE00/58).

## PATIENT CONSENT

All participants gave written informed consent to participate in the study before taking part.

## Supporting information

Supporting Information S1

Supporting Information S2

## Data Availability

Data are available from the corresponding author upon reasonable request.
