# Supplementary material for: Are neuropsychiatric symptoms a marker of small vessel disease progression in older adults? Evidence from the Lothian Birth Cohort 1936
Source: Int J Geriatr Psychiatry. 2022 Dec 9;38(1):e5855. doi: 10.1002/gps.5855 (PMC10108049; doi:10.1002/gps.5855)

**White matter hyperintensity progression and neuropsychiatric symptom course in the Lothian Birth Cohort 1936**

**Supplementary data**

Online supplemental Table 1. Longitudinal imaging studies assessing neuropsychiatric and cognitive symptoms and WMH

Online supplemental Table 2. Baseline characteristics of individuals who (a) did vs did not have MRI at wave two and (b) attended wave two but not wave four MRI

Online supplemental Table 3. Depression: cross-sectional linear models at waves 2/3/4

Online supplemental Table 4. Linear mixed-effects model: Factors associated with depression scores across three waves

Online supplemental Table 5. Linear mixed-effects model: Factors associated with depression scores across three waves including apathy (n=233)

Online supplemental Table 6. Anxiety: cross-sectional linear models at waves 2/3/4

Online supplemental Table 7. Linear mixed-effects model: Factors associated with anxiety scores across three waves

Online supplemental Table 8. Generalised linear models: change in scores at waves two to four - associations with subjective memory concerns at wave 4

Online supplemental Table 9. Apathy subscale analysis

Online supplemental Figure 1. Distribution of change in WMH volumes, anxiety, depression, MMSE, and disability scores across between waves two and four

Online supplemental Figure 2. WMH volume per individual participant at waves 2/3/4 according to quintile of WMH volume change

Online supplemental Figure 3 (a) Linear models: factors associated with depression per wave (b) Linear mixed-effects model plots; factors associated with depression per individual longitudinally across three waves

Online supplemental Figure 4. (a) Linear models: factors associated with anxiety per wave (b) Linear mixed-effects model plots: factors associated with anxiety per individual longitudinally across three waves

Online supplemental Figure 5. Mean mood scores: (a) anxiety (b) depression, according to WMH change quintile at waves 2/3/4

Online supplemental Figure 6. Cross-sectional associations with total apathy scores at wave 4, n=197

Online supplemental Figure 7. Linear model: change scores between waves two to four associated with subjective memory concerns at wave 4

Online supplemental Figure 8. Linear mixed-effects model for WMH associations at three waves with vascular risk factors (including stroke) shown: (a) together (b) separately

Online supplemental Figure 9. Quintile of apathy subscale (Q5=highest apathy scores; Q1=lowest apathy scores) according to WMH volume at each wave

**Online supplemental** **Table 1. Longitudinal imaging studies assessing neuropsychiatric and cognitive symptoms and WMH**

| **Author/year/study** | **N** | **Population/age** | **Follow-up duration** | **Symptom scale used** | **WMH measure used** | **Findings** |
| --- | --- | --- | --- | --- | --- | --- |
| **Depression** | | | | | | |
| van Sloten et al 2015  AGES-Reykyavik study | 1949 | 66-96 year old, free of dementia, without baseline depressive symptoms | Mean 5.2 years | GDS | WMH volumes plus other cSVD markers | Most markers of progression of CSVD over time a/w concurrently developing new depressive symptoms. |
| Godin et al 2008 3C study | 1214 | >65years old | 4 years | CES-D was used but outcome for longitudinal analysis was ‘lifetime major depression’ | WMH volumes | Increase in WMH volume was significantly higher in subjects with baseline lifetime major depression |
| Versluis et al 2006 PROSPER study | 527 | >70s at risk of cardiovascular disease | 33 months | GDS | WMH volumes | WMH were not a/w the development of depressive symptoms |
| Taylor et al 2003 | 133 | Depressed subjects >60 years | 2 years | MADRS ≤ 8 vs MADRS >8 | WMH volumes | Greater progression of WMH a/w poor outcomes in geriatric depression |
| **Subjective memory complaints** | | | | | | |
| Stewart et al 2011 | 1337 | community cohort without dementia, mean age 72.4 | 4 years | Positive response to both: (a) habitual forgetfulness during daily activities and (b) difficulties remembering recent new information. | Per quintile change in total WMH volumes | SMCs at 4years a/w preceding deep subcortical WMH volume quintile change, but not total or periventricular WMH volumes |
| **The Neuropsychiatric Inventory (global score of neuropsychiatric symptom burden)** | | | | | | |
| Kan et al 2020 | 170 | Normal or mildly impaired cognition attending memory clinic, mean age 69.5 | 2 years | Total NPI score | Presence vs absence of WMH progression (Modified Rotterdam Progression visual Scale) | Presence vs absence of  WMH progression associated with increasing total NPI |

**Online supplemental Table 2. Baseline characteristics of individuals who (a) did vs did not have MRI at wave two and (b) attended wave two but not wave four MRI**

|  | **MRI wave 2 (n=672)** | **No MRI wave 2 (n=193)** | **Test, p value** | **(b) Attended wave two MRI but not wave four MRI (n=300)** |
| --- | --- | --- | --- | --- |
| Age (years) Mean (SD) | 72.7 (0.72) | 72.9 (0.66) | t = -1.54, p= 0.12 | 72.7 (0.71) |
| Female | 316 (47.0%) | 101 (52.3%) | X-squared = 1.69,  p = 0.19 | 142 (47.3%) |
| Townsend's Disability Scale score Median [Q1, Q3] | 0 [0, 1] | 0 [0, 1] | W=61600, p = 0.23 | 0 [0, 1] |
| Vascular risk factor score Median [Q1, Q3] | 2 [1, 2] | 2 [1, 2] | W=65663, p = 0.78 | 2 [1, 3] |
| MMSE score Median [Q1, Q3] | 29 [28, 30] | 29 [28, 30] | W=68974, p = 0.14 | 29 [28, 30] |
| HADS Anxiety score Median [Q1, Q3] | 4 [2, 6] | 5 [2, 7] | W=61989, p = 0.34 | 4 [2, 7] |
| HADS Depression score Median [Q1, Q3] | 2 [1, 4] | 2 [1, 4] | W=64469, p = 0.89 | 2 [1, 4] |
| WMH volume (ml) Mean (SD) | 12.1 (12.8) | - | - | 13.2 (14.0) |
| WMH volume (ml) Median [Q1, Q3] | 7.70 [3.65, 17.0] | - | - | 8.22 [3.88, 19.2] |

**Online supplemental** **Table 3. Depression: cross-sectional linear models at waves 2/3/4 with depression at each timepoint as the outcome.** Adjusted for age, sex, MMSE score, Anxiety score, contemporaneous Normalised WMH volume:ICV at relevant timepoint, Vascular risk factor score, years of education, Disability score, and apathy score at wave 4

|  | | | **Depression wave 2** | | | | **Depression wave 3** | | | | **Depression wave 4** | | | |
| --- | --- | --- | --- | --- | --- | --- | --- | --- | --- | --- | --- | --- | --- | --- |
| *Predictors* | *std. Beta* | *standardized CI* | | | *p* | *std. Beta* | *standardized CI* | | *p* | *std. Beta* | | | *standardized CI* | *p* |
| (Intercept) | 0.14 | 0.05 – 0.23 | | | 0.381 | 0.13 | 0.02 – 0.23 | | 0.501 | -0.04 | | | -0.19 – 0.11 | 0.857 |
| Age (years) | 0.04 | -0.03 – 0.11 | | | 0.253 | 0.05 | -0.03 – 0.12 | | 0.246 | -0.02 | | | -0.12 – 0.09 | 0.748 |
| Sex: Female | -0.30 | -0.44 – -0.16 | | | <0.001 | -0.28 | -0.44 – -0.12 | | 0.001 | 0.08 | | | -0.14 – 0.31 | 0.475 |
| MMSE | -0.02 | -0.10 – 0.05 | | | 0.489 | -0.14 | -0.22 – -0.05 | | 0.001 | 0.10 | | | -0.01 – 0.21 | 0.080 |
| Anxiety | 0.36 | 0.29 – 0.43 | | | <0.001 | 0.33 | 0.25 – 0.41 | | <0.001 | 0.35 | | | 0.24 – 0.46 | <0.001 |
| Normalised WMH volume: ICV | -0.03 | -0.09 – 0.04 | | | 0.476 | 0.07 | -0.00 – 0.15 | | 0.065 | -0.02 | | | -0.13 – 0.09 | 0.672 |
| Vascular risk factors | 0.06 | -0.01 – 0.13 | | | 0.082 | 0.03 | -0.04 – 0.11 | | 0.404 | 0.10 | | | -0.00 – 0.21 | 0.057 |
| Years of education | 0.02 | -0.05 – 0.09 | | | 0.496 | 0.03 | -0.05 – 0.11 | | 0.457 | -0.04 | | | -0.15 – 0.07 | 0.467 |
| Townsend's Disability Scale score | 0.26 | 0.19 – 0.33 | | | <0.001 | 0.31 | 0.23 – 0.39 | | <0.001 | 0.21 | | | 0.10 – 0.32 | <0.001 |
| Apathy score total | - | - | | | - | - | - | | - | 0.43 | | | 0.31 – 0.55 | <0.001 |
| Observations | | | | 669 | | | | 469 | | | | 197 | | |
| R^2^ / R^2^ adjusted | | | | 0.227 / 0.218 | | | | 0.306 / 0.294 | | | | 0.470 / 0.444 | | |

**Online supplemental Table 4. Linear mixed-effects model: Factors associated with depression scores across three waves** HADS Depression score at Waves 2/3/4 as outcome; model accounts for Wave 2/3/4 (Time), baseline age, sex, MMSE, HADS Anxiety scores, Normalised WMH volumes:ICV, baseline vascular risk factors, Townsend Disability scale scores, years of full-time education

|  | **Depression** | | |
| --- | --- | --- | --- |
| *Predictors* | *std. Beta* | *95% CI* | *p* |
| (Intercept) | 0.13 | 0.04 – 0.21 | 0.310 |
| Wave | -0.10 | -0.31 – 0.10 | 0.327 |
| Age (years) | 0.16 | -0.05 – 0.38 | 0.130 |
| Sex: Female | -0.26 | -0.38 – -0.15 | <0.001 |
| MMSE score | -0.05 | -0.09 – -0.01 | 0.026 |
| HADS Anxiety score | 0.32 | 0.27 – 0.37 | <0.001 |
| Normalised WMH volume: ICV | -0.01 | -0.06 – 0.05 | 0.783 |
| Vascular risk factors | 0.05 | 0.00 – 0.10 | 0.050 |
| Townsend Disability Scale score | 0.24 | 0.20 – 0.29 | <0.001 |
| Full-time education (years) | 0.01 | -0.05 – 0.07 | 0.685 |
| **Random Effects** | | | |
| σ^2^ | 1.63 | | |
| τ_00_ _lbc36no_ | 2.10 | | |
| ICC | 0.56 | | |
| N _lbc36no_ | 688 | | |
| Observations | 1517 | | |
| Marginal R^2^ / Conditional R^2^ | 0.205 / 0.653 | | |

**Online supplemental** **Table 5. Linear mixed-effects model: Factors associated with depression scores across three waves including Wave 4 apathy score (n=233)** HADS Depression score at Waves 2/3/4 as outcome; model accounts for Wave 2/3/4 (Time), baseline age, sex, MMSE, HADS Anxiety scores, Normalised WMH volumes:ICV, baseline vascular risk factors, Townsend Disability scale scores, years of full-time education, Wave 4 apathy score

|  | **Depression** | | |
| --- | --- | --- | --- |
| *Predictors* | *std. Beta* | *standardized CI* | *p* |
| (Intercept) | 0.01 | -0.12 – 0.13 | 0.582 |
| Wave | 0.23 | -0.17 – 0.63 | 0.261 |
| Age (years) | -0.15 | -0.56 – 0.25 | 0.463 |
| Sex: Female | 0.03 | -0.16 – 0.21 | 0.756 |
| MMSE | -0.02 | -0.08 – 0.04 | 0.565 |
| Anxiety | 0.32 | 0.25 – 0.40 | <0.001 |
| Normalised WMH volume: ICV | 0.00 | -0.09 – 0.09 | 0.992 |
| Vascular risk factors | 0.04 | -0.04 – 0.12 | 0.327 |
| Townsend's Disability Scale score | 0.18 | 0.11 – 0.25 | <0.001 |
| Years of education | 0.06 | -0.03 – 0.15 | 0.170 |
| Apathy score total | 0.34 | 0.25 – 0.44 | <0.001 |
| **Random Effects** | | | |
| σ^2^ | 1.69 | | |
| τ_00_ _lbc36no_ | 1.61 | | |
| ICC | 0.49 | | |
| N _lbc36no_ | 233 | | |
| Observations | 615 | | |
| Marginal R^2^ / Conditional R^2^ | 0.335 / 0.659 | | |

**Online supplemental** **Table 6. Anxiety: cross-sectional linear models at waves 2/3/4 with anxiety at each timepoint as the outcome.** Adjusted for age, sex, MMSE score, HADS Depression score, contemporaneous Normalised WMH volume:ICV at relevant timepoint, Vascular risk factor score, and years of education

|  | | | | **Anxiety wave 2** | | | | | **Anxiety wave 3** | | | | | **Anxiety wave 4** | | | |  |
| --- | --- | --- | --- | --- | --- | --- | --- | --- | --- | --- | --- | --- | --- | --- | --- | --- | --- | --- |
| *Predictors* | *std. Beta* | | *standardized CI* | | | *p* | *std. Beta* | | *standardized CI* | | *p* | *std. Beta* | | | *standardized CI* | | *p* |  |
| (Intercept) | -0.19 | | -0.29 – -0.09 | | | 0.194 | -0.15 | | -0.26 – -0.04 | | 0.005 | -0.15 | | | -0.28 – -0.03 | | 0.229 |  |
| Age (years) | -0.01 | | -0.09 – 0.06 | | | 0.692 | -0.11 | | -0.19 – -0.02 | | 0.012 | -0.05 | | | -0.14 – 0.05 | | 0.335 |  |
| Sex: Female | 0.40 | | 0.26 – 0.55 | | | <0.001 | 0.32 | | 0.15 – 0.49 | | <0.001 | 0.33 | | | 0.14 – 0.52 | | 0.001 |  |
| MMSE | -0.11 | | -0.18 – -0.03 | | | 0.004 | 0.00 | | -0.08 – 0.09 | | 0.927 | 0.01 | | | -0.09 – 0.11 | | 0.872 |  |
| Depression | 0.37 | | 0.30 – 0.44 | | | <0.001 | 0.38 | | 0.29 – 0.46 | | <0.001 | 0.37 | | | 0.28 – 0.47 | | <0.001 |  |
| Normalised WMH volume: ICV | 0.01 | | -0.06 – 0.09 | | | 0.707 | 0.04 | | -0.05 – 0.12 | | 0.394 | -0.01 | | | -0.11 – 0.08 | | 0.824 |  |
| Vascular risk factors | 0.01 | | -0.06 – 0.08 | | | 0.798 | 0.05 | | -0.03 – 0.13 | | 0.244 | -0.08 | | | -0.17 – 0.02 | | 0.114 |  |
| Years of education | -0.02 | | -0.09 – 0.05 | | | 0.624 | -0.09 | | -0.17 – -0.00 | | 0.044 | -0.07 | | | -0.16 – 0.03 | | 0.176 |  |
| Observations | | 670 | | |  | | | 470 | |  | | | 380 | | |  | | |
| R^2^ / R^2^ adjusted | | 0.181 / 0.172 | | |  | | | 0.191 / 0.178 | |  | | | 0.165 / 0.149 | | |  | | |

**Online supplemental** **Table 7. Linear mixed-effects model: Factors associated with anxiety scores across three waves** HADS Anxiety score at Waves 2/3/4 as outcome; model accounts for Wave 2/3/4 (Time), baseline age, sex, MMSE, HADS Depression scores, Normalised WMH volumes:ICV, baseline vascular risk factors, years of full-time education.

|  | **Anxiety** | | |
| --- | --- | --- | --- |
| *Predictors* | *std. Beta* | *standardized CI* | *p* |
| (Intercept) | -0.15 | -0.23 – -0.06 | 0.818 |
| Wave | -0.12 | -0.33 – 0.10 | 0.278 |
| Age (years) | 0.04 | -0.18 – 0.26 | 0.745 |
| Sex: Female | 0.35 | 0.22 – 0.48 | <0.001 |
| MMSE | 0.00 | -0.04 – 0.04 | 0.984 |
| Depression | 0.30 | 0.26 – 0.35 | <0.001 |
| Normalised WMH volume: ICV | 0.05 | -0.01 – 0.10 | 0.134 |
| Vascular risk factors | 0.00 | -0.05 – 0.06 | 0.872 |
| Years of education | -0.06 | -0.13 – 0.00 | 0.053 |
| **Random Effects** | | | |
| σ^2^ | 2.80 | | |
| τ_00_ _lbc36no_ | 5.13 | | |
| ICC | 0.65 | | |
| N _lbc36no_ | 689 | | |
| Observations | 1520 | | |
| Marginal R^2^ / Conditional R | 0.134 / 0.694 | | |

**Online supplemental** **Table 8. Generalised linear models: change in scores at waves 2-4 associations with subjective memory concerns at wave 4 .** This statistical approach was used as SMC was only measured at wave 4**.** Adjusted for age, sex, MMSE score, HADS Depression score change, Baseline depression Score, HADS Anxiety score change, MMSE score change, Normalised WMH volume:ICV change, Baseline Normalised WMH volume:ICV, baseline Vascular risk factor score, and years of education.

| **Do you currently have any problems with your memory?** | | | | **Do you forget where you have left things more often than you used to?** | | |  |  |
| --- | --- | --- | --- | --- | --- | --- | --- | --- |
| *Predictors* | *Odds Ratios* | *CI* | *p* | *Odds Ratios* | *CI* | *p* |  |  |
| (Intercept) | 0.07 | 0.00 – 329268093158.79 | 0.857 | 0.01 | 0.00 – 14285885189.24 | 0.739 |  |  |
| Age (years) | 1.03 | 0.71 – 1.48 | 0.877 | 1.07 | 0.75 – 1.52 | 0.712 |  |  |
| Sex: Female | 1.02 | 0.65 – 1.60 | 0.924 | 0.86 | 0.56 – 1.33 | 0.502 |  |  |
| Depression score change | 1.27 | 1.11 – 1.48 | 0.001 | 1.12 | 0.99 – 1.26 | 0.079 |  |  |
| Baseline Depression score | 1.27 | 1.11 – 1.47 | 0.001 | 1.14 | 1.02 – 1.29 | 0.025 |  |  |
| Anxiety score change | 1.00 | 0.91 – 1.09 | 0.993 | 1.07 | 0.98 – 1.17 | 0.140 |  |  |
| MMSE score change | 0.92 | 0.81 – 1.04 | 0.193 | 0.94 | 0.83 – 1.05 | 0.274 |  |  |
| Normalised WMH volume: ICV change | 1.12 | 0.55 – 2.26 | 0.758 | 1.24 | 0.63 – 2.44 | 0.530 |  |  |
| Baseline Normalised WMH volume: ICV | 1.02 | 0.52 – 1.99 | 0.957 | 1.05 | 0.55 – 2.00 | 0.878 |  |  |
| Vascular risk factors | 0.92 | 0.75 – 1.12 | 0.402 | 0.95 | 0.78 – 1.16 | 0.632 |  |  |
| Years of education | 1.01 | 0.84 – 1.23 | 0.891 | 0.89 | 0.74 – 1.07 | 0.232 |  |  |
| Observations | 358  R^2^ Tjur 0.056 | | | Observations 355  R^2^ Tjur 0.047 | | |  | 355 |

**Online supplemental** **Table 9. Apathy subscale analysis (a) Executive subscale (b) Emotional subscale (c) Initiation subscale**

Normalised WMH volume:ICV at Waves 2/3/4 as outcome; model accounts for Wave 2/3/4 (Time), baseline age, sex, MMSE, baseline vascular risk factors, years of full-time education, HADS Anxiety scores, HADS Depression scores, Apathy subscales, Subjective memory complaints Q1 at wave 4 , Townsend’s Disability Scale Score

| **Normalised WMH volume:ICV** | | | | | | | | | |
| --- | --- | --- | --- | --- | --- | --- | --- | --- | --- |
| *Predictors* | *std. Beta* | *standardized CI* | *p* | *std. Beta* | *standardized CI* | *p* | *std. Beta* | *standardized CI* | *p* |
| (Intercept) | 0.020 | -0.190 – 0.230 | 0.023 | -0.025 | -0.229 – 0.180 | 0.022 | -0.020 | -0.226 – 0.185 | 0.021 |
| WAVE | -0.182 | -0.435 – 0.071 | 0.158 | -0.172 | -0.425 – 0.081 | 0.183 | -0.173 | -0.426 – 0.080 | 0.181 |
| Age (years) | 0.414 | 0.157 – 0.671 | 0.002 | 0.403 | 0.146 – 0.660 | 0.002 | 0.404 | 0.147 – 0.661 | 0.002 |
| MMSE | -0.029 | -0.063 – 0.004 | 0.088 | -0.029 | -0.063 – 0.004 | 0.086 | -0.029 | -0.063 – 0.004 | 0.087 |
| Sex | 0.019 | -0.106 – 0.144 | 0.770 | 0.041 | -0.088 – 0.170 | 0.533 | 0.023 | -0.103 – 0.150 | 0.715 |
| Vascular risk factors | -0.005 | -0.060 – 0.050 | 0.862 | -0.005 | -0.060 – 0.050 | 0.860 | -0.007 | -0.062 – 0.048 | 0.793 |
| Years of education | -0.021 | -0.147 – 0.106 | 0.750 | -0.016 | -0.141 – 0.110 | 0.806 | 0.003 | -0.123 – 0.130 | 0.958 |
| Anxiety | 0.022 | -0.024 – 0.069 | 0.352 | 0.028 | -0.018 – 0.075 | 0.234 | 0.027 | -0.019 – 0.074 | 0.250 |
| Depression score | -0.025 | -0.070 – 0.019 | 0.260 | -0.023 | -0.067 – 0.021 | 0.304 | -0.025 | -0.069 – 0.019 | 0.272 |
| Apathy: subscale a/b/c | 0.130 | -0.002 – 0.261 | 0.053 | 0.133 | 0.004 – 0.262 | 0.044 | 0.110 | -0.016 – 0.237 | 0.087 |
| Subjective memory problems: Yes | -0.015 | -0.286 – 0.256 | 0.913 | 0.061 | -0.198 – 0.320 | 0.644 | 0.052 | -0.208 – 0.312 | 0.694 |
| Townsend's Disability Scale score | -0.007 | -0.049 – 0.035 | 0.741 | -0.005 | -0.046 – 0.037 | 0.830 | -0.006 | -0.047 – 0.036 | 0.791 |

| **Random Effects** | |  |  | |  | |  | |
| --- | --- | --- | --- | --- | --- | --- | --- | --- |
| σ^2^ | 0.03 | σ^2^ | | 0.03 | | σ^2^ | | 0.03 |
| τ_00_ _lbc36no_ | 0.45 | τ_00_ _lbc36no_ | | 0.45 | | τ_00_ _lbc36no_ | | 0.45 |
| ICC | 0.93 | ICC | | 0.93 | | ICC | | 0.93 |
| N _lbc36no_ | 226 | N _lbc36no_ | | 226 | | N _lbc36no_ | | 226 |
| Observations | 596 | Observations | | 596 | | Observations | | 596 |
| Marginal R^2^/Conditional R^2^ | 0.070 / 0.934 | Marginal R^2^/Conditional R^2^ | | 0.073 / 0.934 | | Marginal R^2^/Conditional R^2^ | | 0.068 / 0.934 |

**Online supplemental** **Figure 1. Distribution of change in WMH volumes, anxiety, depression, MMSE, and disability scores between wave two and wave four** Positive values denote increase; negative values denote decrease; value of 0 denotes no chnage between wave two and wave four


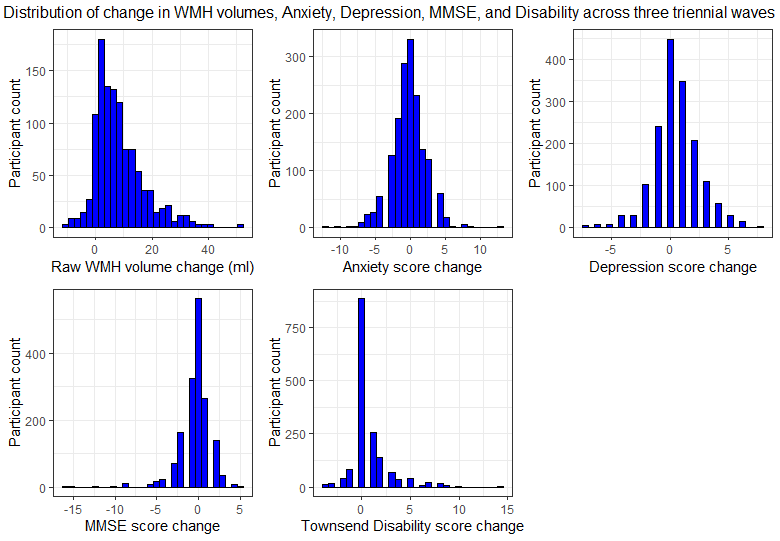


**Online supplemental Figure 2. WMH volume per individual participant at waves 2/3/4 according to quintile of WMH volume change**


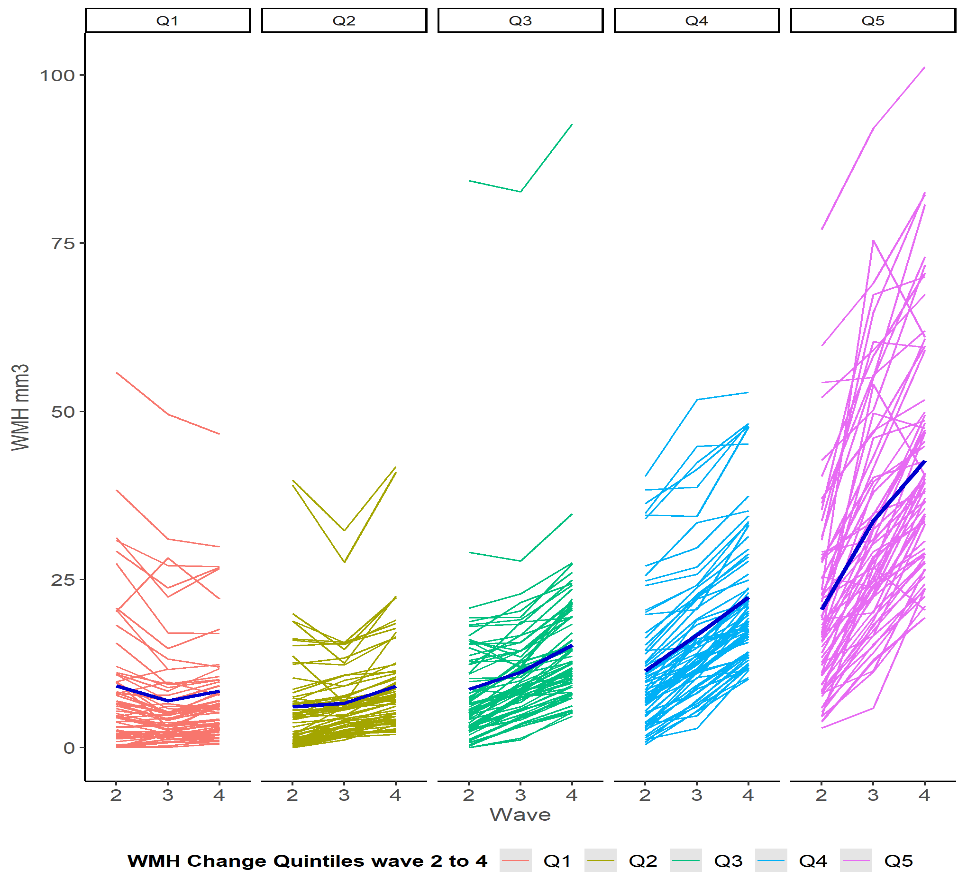


**Online supplemental** **Figure 3. Linear models: factors associated with depression (a) at each of waves 2, 3 and 4 (b) Linear mixed-effects model plots: factors associated with depression per individual participant incorporating longitudinal data across all three waves.** Positive estimates (blue) = positive relationships with depression; negative estimates (red) = negative relationships with depression

**(a)**


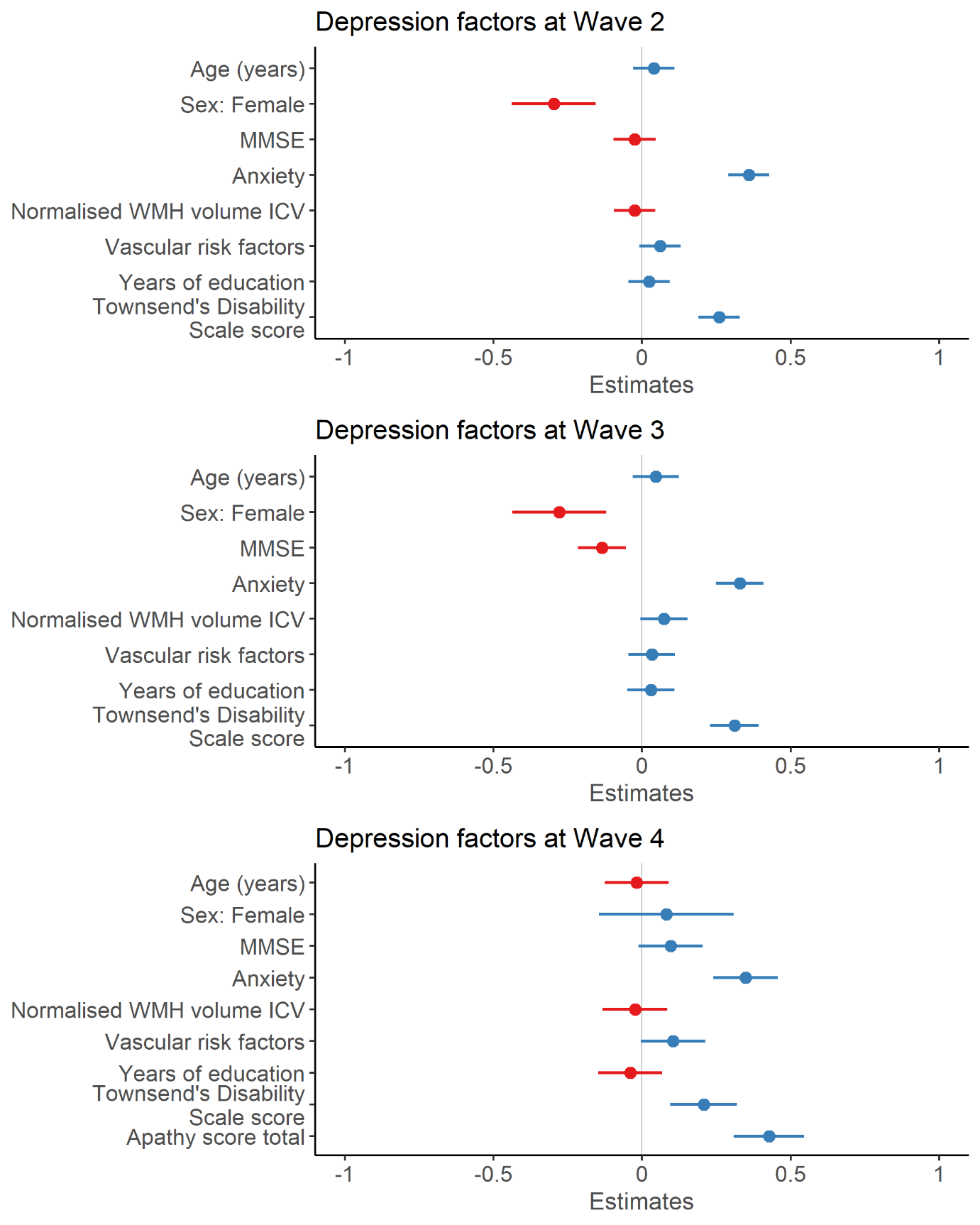


**(b)**

**
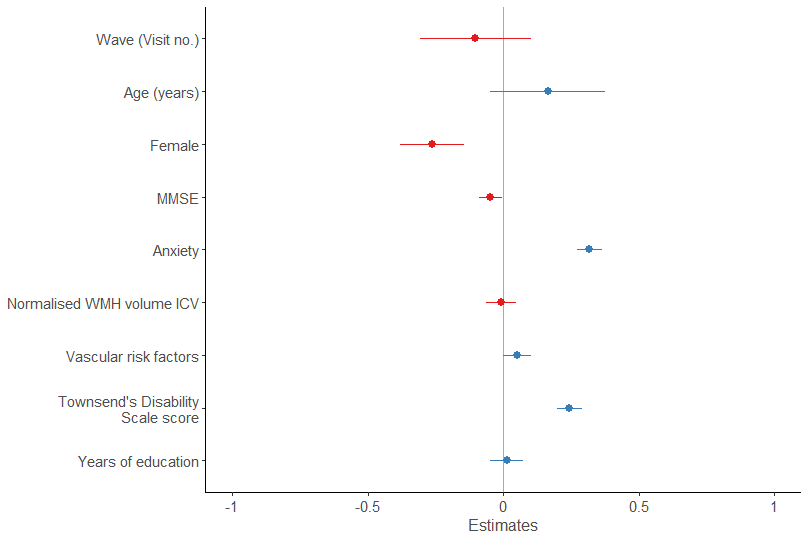
**

**Online supplemental** **Figure 4. (a) Linear models: factors associated with anxiety per wave (b) (b) Linear mixed-effects model plots: factors associated with anxiety per individual participant incorporating longitudinal data across all three waves.** Positive estimates (blue) = positive relationships with anxiety; negative estimates (red) = negative relationships with anxiety

**(a)**

**
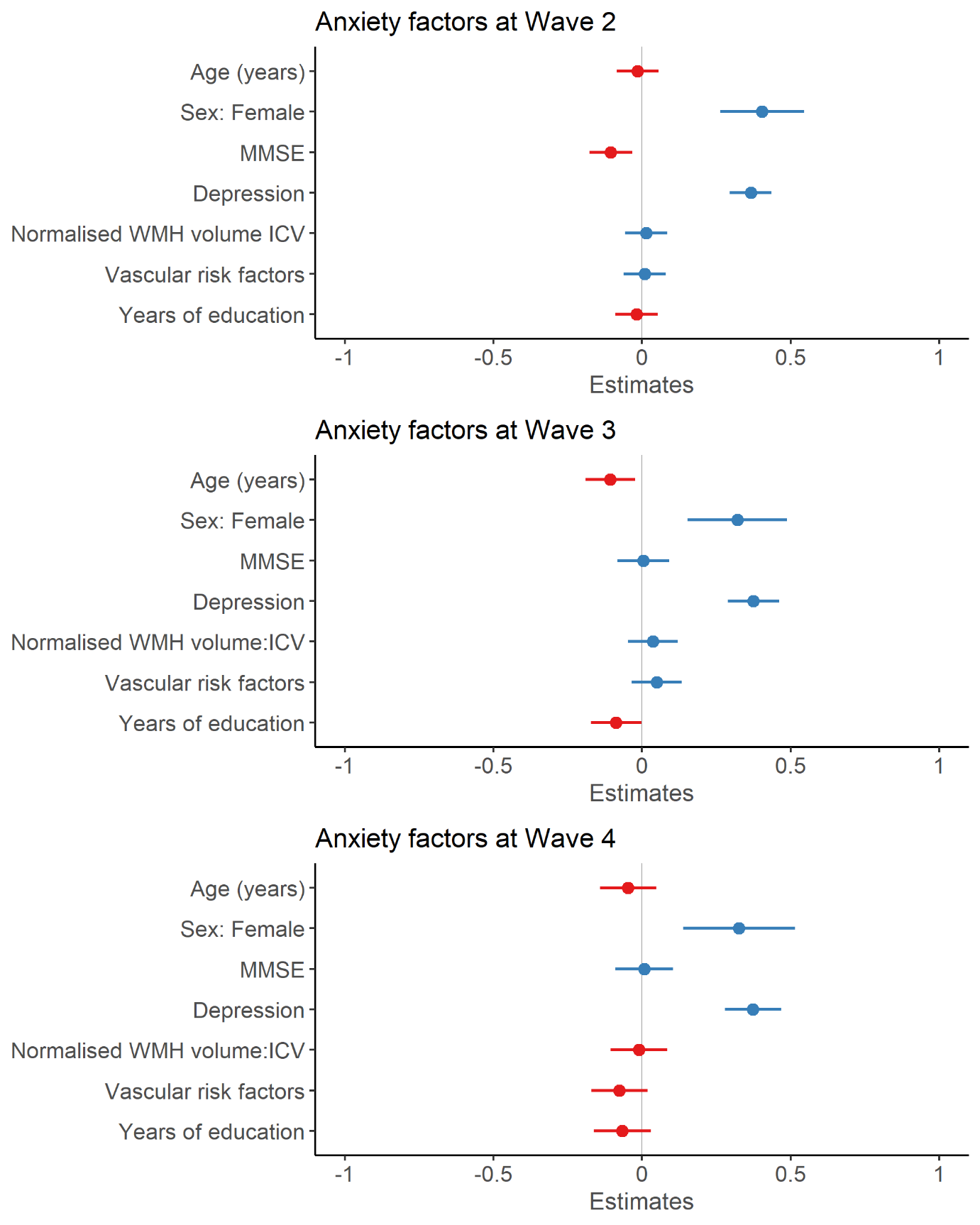
**

**(b)
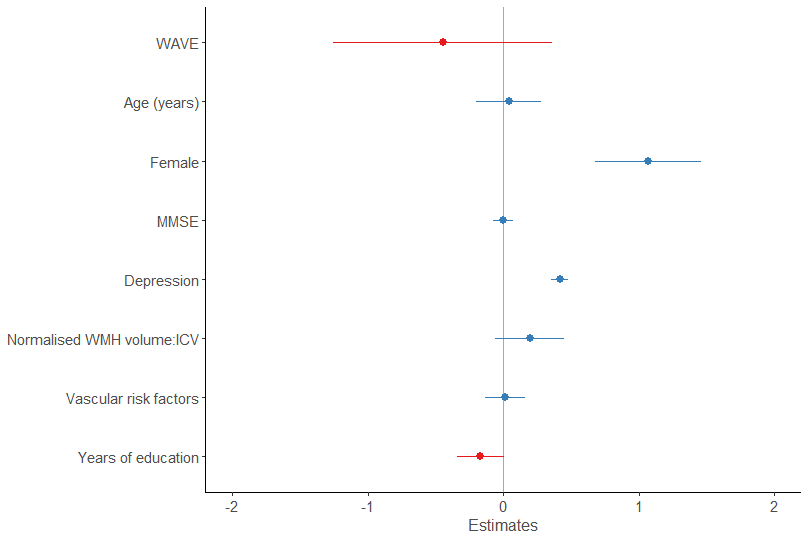
**

**Online supplemental Figure 5. Mean mood scores: (a) anxiety (b) depression, according to WMH change quintile at waves 2/3/4** Q1=greatest reduction in WMH volume; Q5=greatest increase in WMH volume

**
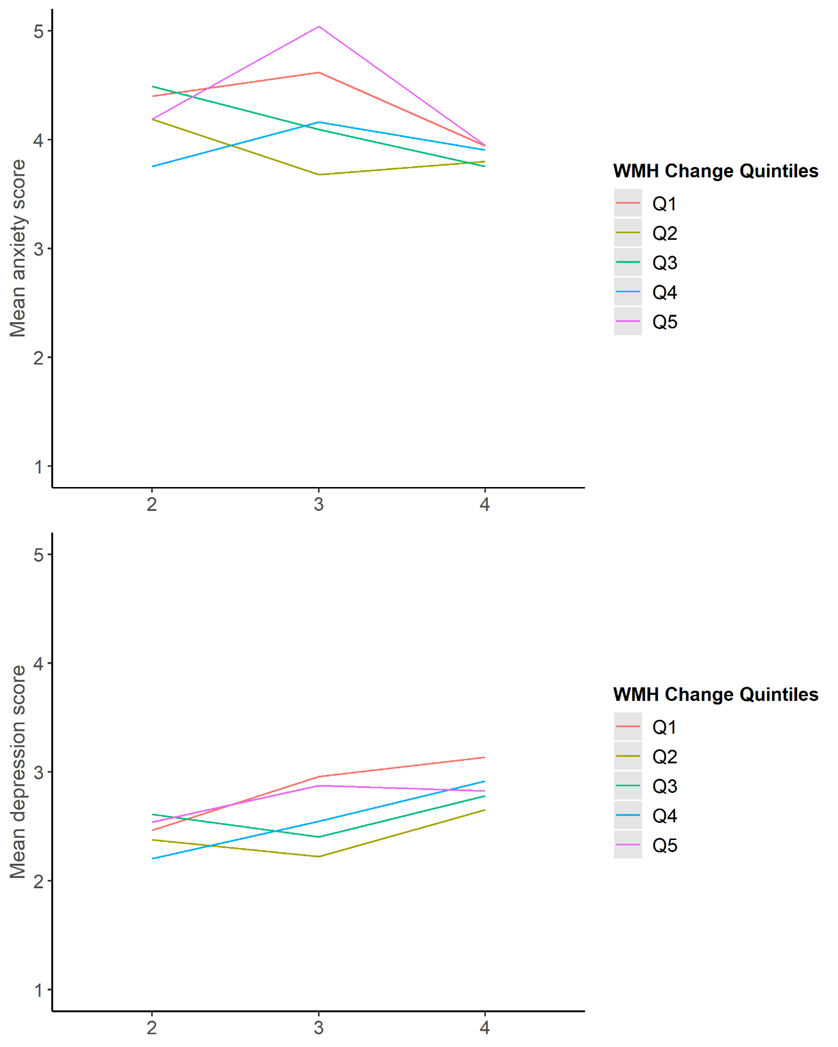
**

**Online supplemental** **Figure 6. Cross-sectional associations with total apathy scores at wave** **4 as the oucome, n=197.** Adjusted for age, sex, HADS Depression score, MMSE score, contemporaneous Normalised WMH volume:ICV at wave 4, Vascular risk factor score, years of education and Disability score.


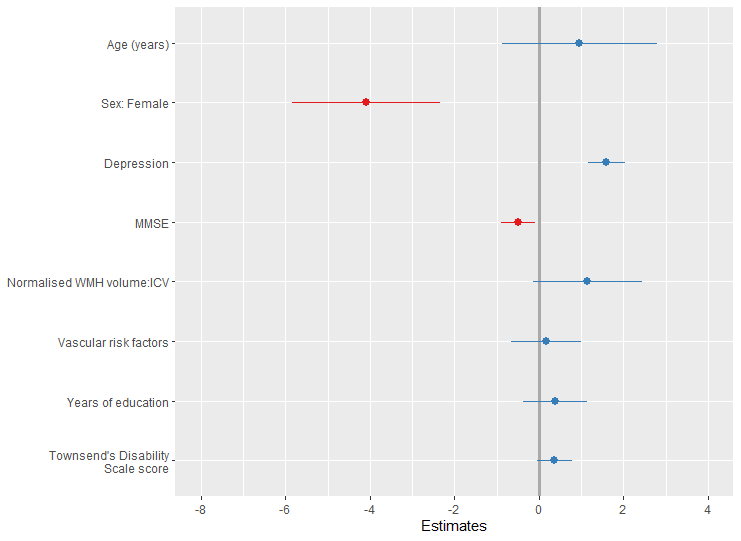


**Online supplemental** **Figure 7. Linear model: change scores between waves two to four associated with subjective memory concerns at wave 4 as the outcome**

Adjusted for age, sex, baseline HADS Depression score, HADS Anxiety score change, MMSE score change, Normalised WMH volume:ICV change, baseline Normalised WMH volume:ICV, Vascular risk factor score, years of education.


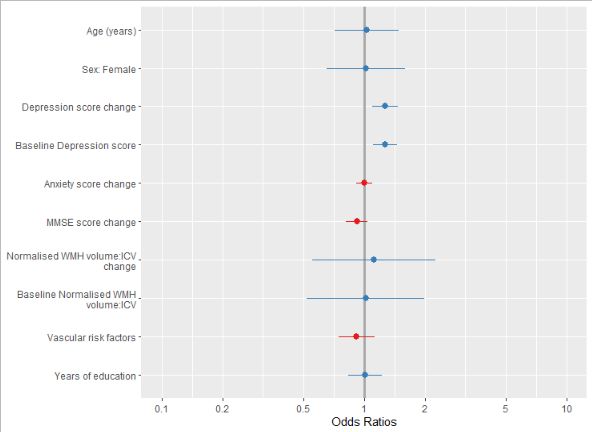


**Online supplemental** **Figure 8. Linear mixed-effects model for WMH change across three waves associations with all neuropsychiatric symptoms, with vascular risk factor covariate shown (a) as sum score (b) separately** WMH/ICV at waves 2/3/4 = outcome; anxiety, depression, apathy, subjective memory complains, adjusted for age, sex, MMSE, vascular risk factors, Townsend’s Disability Scale score, years of education


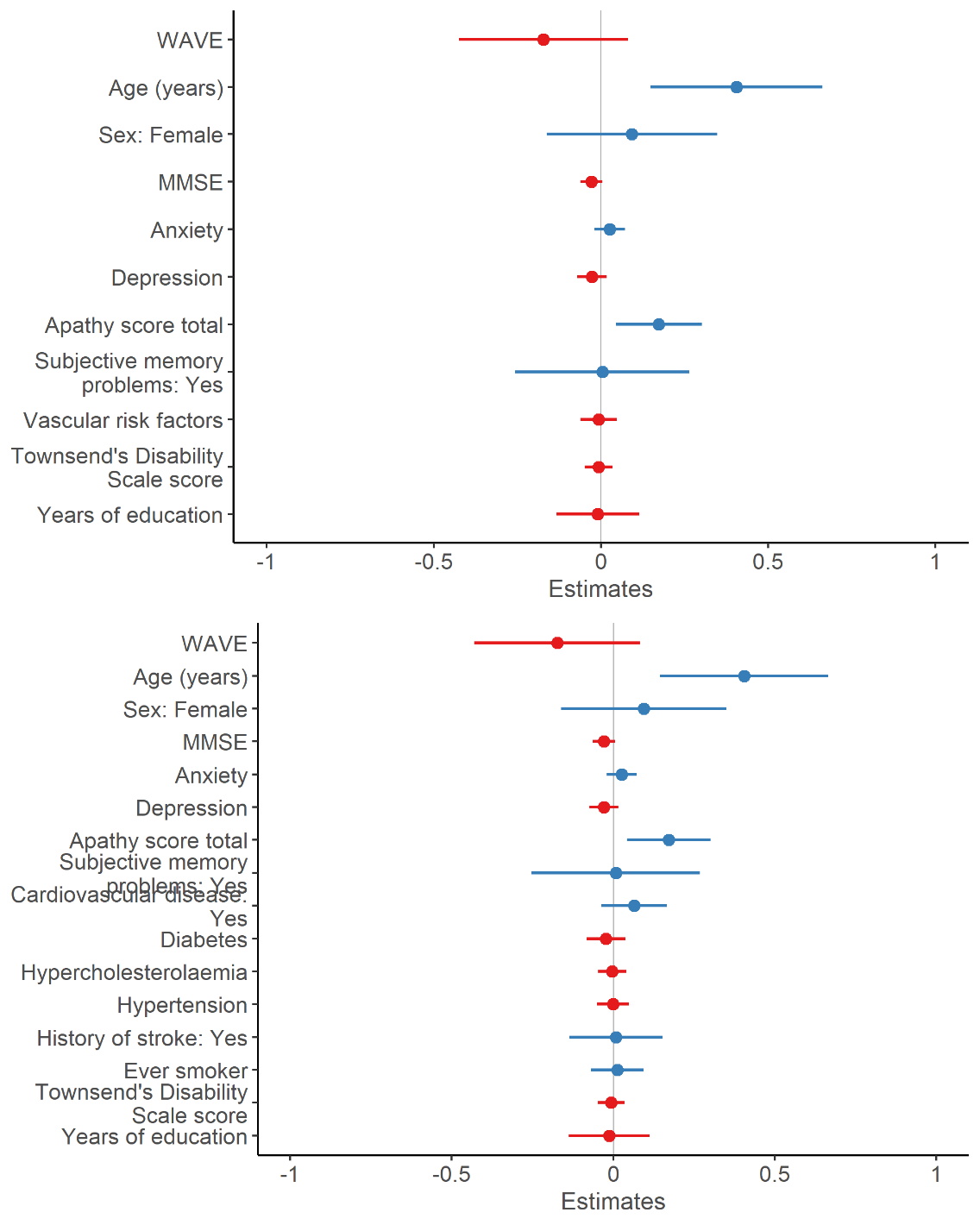


**Online supplemental** **Figure 9. Quintile of apathy subscale (Q5=highest apathy scores; Q1=lowest apathy scores) according to WMH volume at each wave**


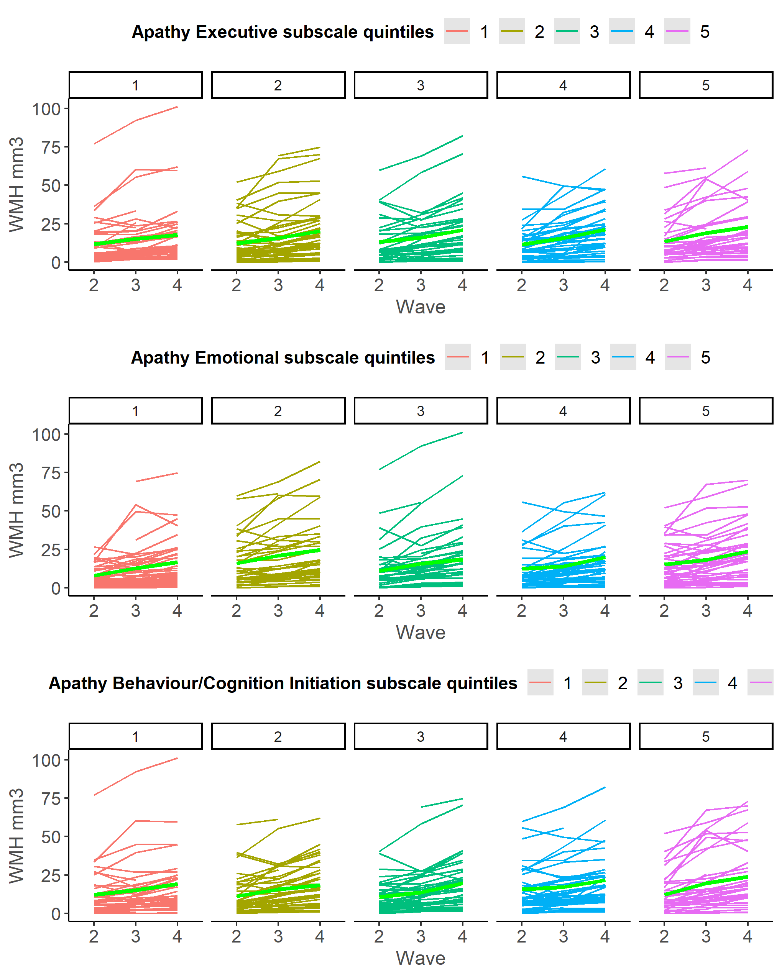

Supplement: Supplementary file 1 — Supporting Information S1 [file GPS-38-0-s002.docx]
